# Breast cancer classification in point-of-care ultrasound imaging—the impact of training data

**DOI:** 10.1117/1.JMI.12.1.014502

**Published:** 2025-01-17

**Authors:** Jennie Karlsson, Ida Arvidsson, Freja Sahlin, Kalle Åström, Niels Christian Overgaard, Kristina Lång, Anders Heyden

**Affiliations:** a Lund University, Centre for Mathematical Sciences, Division of Computer Vision and Machine Learning, Lund, Sweden; b Lund University, Division of Diagnostic Radiology, Department of Translational Medicine, Lund, Sweden; c Skåne University Hospital, Unilabs Mammography Unit, Malmö, Sweden

**Keywords:** breast ultrasound, point-of-care ultrasound, breast cancer, cycle-consistent adversarial networks, convolutional neural networks

## Abstract

**Purpose:**

The survival rate of breast cancer for women in low- and middle-income countries is poor compared with that in high-income countries. Point-of-care ultrasound (POCUS) combined with deep learning could potentially be a suitable solution enabling early detection of breast cancer. We aim to improve a classification network dedicated to classifying POCUS images by comparing different techniques for increasing the amount of training data.

**Approach:**

Two data sets consisting of breast tissue images were collected, one captured with POCUS and another with standard ultrasound (US). The data sets were expanded by using different techniques, including augmentation, histogram matching, histogram equalization, and cycle-consistent adversarial networks (CycleGANs). A classification network was trained on different combinations of the original and expanded data sets. Different types of augmentation were investigated and two different CycleGAN approaches were implemented.

**Results:**

Almost all methods for expanding the data sets significantly improved the classification results compared with solely using POCUS images during the training of the classification network. When training the classification network on POCUS and CycleGAN-generated POCUS images, it was possible to achieve an area under the receiver operating characteristic curve of 95.3% (95% confidence interval 93.4% to 97.0%).

**Conclusions:**

Applying augmentation during training showed to be important and increased the performance of the classification network. Adding more data also increased the performance, but using standard US images or CycleGAN-generated POCUS images gave similar results.

## 1 Introduction

Globally, breast cancer is the most predominant type of cancer affecting women. The survival rate is suffering from inequity where it is considerably lower for women in low- and middle-income countries (LMICs) compared with that for women in high-income countries (HICs). For women in HICs, the survival rate is ∼90%, but it decreases to 66% in India and 40% in South Africa.^1^

A contributing factor to the poor survival in LMICs is the limited access to early diagnosis, resulting in women often having advanced-stage disease at presentation. In 2021, the World Health Organization (WHO) launched the Global Breast Cancer Initiative with the aim to reduce breast cancer mortality by 2.5% per year until 2040.^1^ This would avert 2.5 million breast cancer deaths over a 20-year period. To reach this goal, the WHO laid forward three focus areas, of which one was access to timely diagnosis. The successful implementation of screening programs in HICs is dependent on strong healthcare systems. Enabling timely diagnosis in low-resource settings requires cost-effective, agile diagnostic solutions.^2^

Point-of-care ultrasound (POCUS), a small portable ultrasound (US) device that can fit in a pocket, has gained increasing attention in recent years and has been shown to be a useful diagnostic tool in low-resource settings.^3^ Thanks to recent developments, the technique can now deliver images of good quality and thus shows promise as a candidate method to enable access to timely breast cancer diagnosis where standard high-end US imaging is not available.

As the analysis of breast US images is demanding and requires trained professionals, decision-support using deep learning could enable a wider implementation.^4^ Previous studies using convolutional neural networks (CNNs) for the classification of breast cancer in standard US images have shown promising results.^5^^–^^7^ Furthermore, Love et al.^7^ performed a pilot study exploring the possibility of triaging women with breast symptoms using POCUS in combination with computer-assisted diagnosis. However, the sample size was small, and further studies are needed. To the best of our knowledge, there are no dedicated tools for the analysis of POCUS breast images, and there are no publicly available data sets.

The purpose of this study was to investigate different methods for expanding the training data, with the goal of improving the performance of a classification network dedicated to classifying POCUS breast images. A data set containing POCUS breast images was collected, and techniques including augmentation, histogram matching, histogram equalization, and cycle-consistent adversarial networks (CycleGANs)^8^ were investigated to increase the amount of training data.

CycleGAN has previously been used in medical applications, such as by Wolterink et al.,^9^ where it showed promising results in translating images acquired with magnetic resonance (MR) into the domain of computed tomography (CT). In our study, the CycleGAN algorithm will be used to shift the domain of standard US images into the domain of POCUS images, that is, to generate more data appearing to be collected using POCUS, hence expanding the POCUS data. This was further used in the training of a classification network for breast cancer detection in POCUS images, with the goal of improving its performance.

This work is an extension of Karlsson et al.^10^ presented at SPIE Medical Imaging 2023. In this paper, two different approaches were implemented for the CycleGAN algorithm, compared with the previous study where only the first approach was implemented. In the first approach, a CycleGAN was trained on all data regardless of the classes of the images. In the second approach, three different CycleGANs were trained, one for each class. Furthermore, histogram matching, histogram equalization, and different types of augmentation were investigated. Finally, the CycleGAN and classification network algorithms have been trained on more data compared with the original study, which makes the results more robust.

## 2 Method

**Fig. 1:**
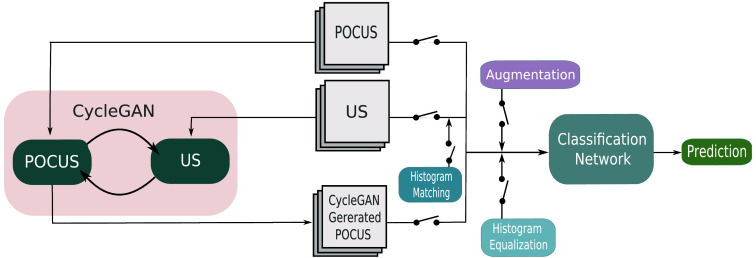
Scheme over the setup for training a classification network on different combinations of data.

In this study, it was investigated if the performance of a classification network could be improved when trained on different combinations of data. The data consisted of combinations of POCUS images, standard US images, POCUS images generated with CycleGAN, images transformed with histogram matching, images normalized with histogram equalization, and images with applied augmentation. Figure 1 displays a scheme over the combinations of data as input to the classification network. In this section, the scheme will be explained in more detail.

### 2.1 Data Sets

Two data sets were used, both consisting of breast US images. Each image is labeled to one of the following three classes: normal, benign, or malignant. The normal class contains images of normal tissue, the benign class contains non-cancerous lesions, and the malignant class contains cancerous lesions. The data sets were collected at the Unilabs Mammography unit at Skåne University Hospital in Malmö, Sweden. The images in the first data set were collected with standard US machines, Logiq E9 and Logiq E10, whereas a POCUS device, Vscan air from GE,^11^ was used for collecting the images of the second data set.

The first data set contained standard US images from 436 individuals and was collected retrospectively between the years 2017 and 2018. The labeling of these images was based on the standard of care and follow up. Lesions were assessed by imaging and if needed, histopathology, according to clinical routine. In negative cases, the label was based on at least one-year follow-up.

The second data set containing POCUS images from 105 individuals was collected prospectively on patients undergoing standard-of-care breast diagnostic assessment, which was also examined with POCUS.

There was no overlap of patients in the two data sets. Images of normal tissue were at times acquired from the contralateral breast for patients with findings.

**Fig. 2:**
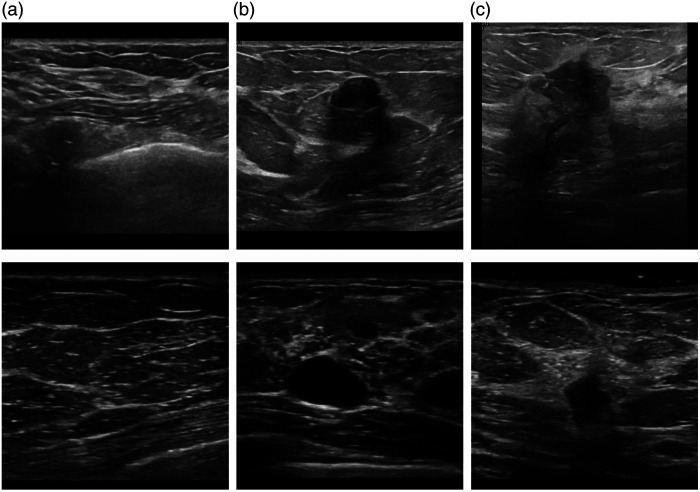
Examples of US images (upper row) and POCUS images (lower row) capturing normal tissue (a), benign lesion (b), and malignant lesion (c).

**Fig. 3:**
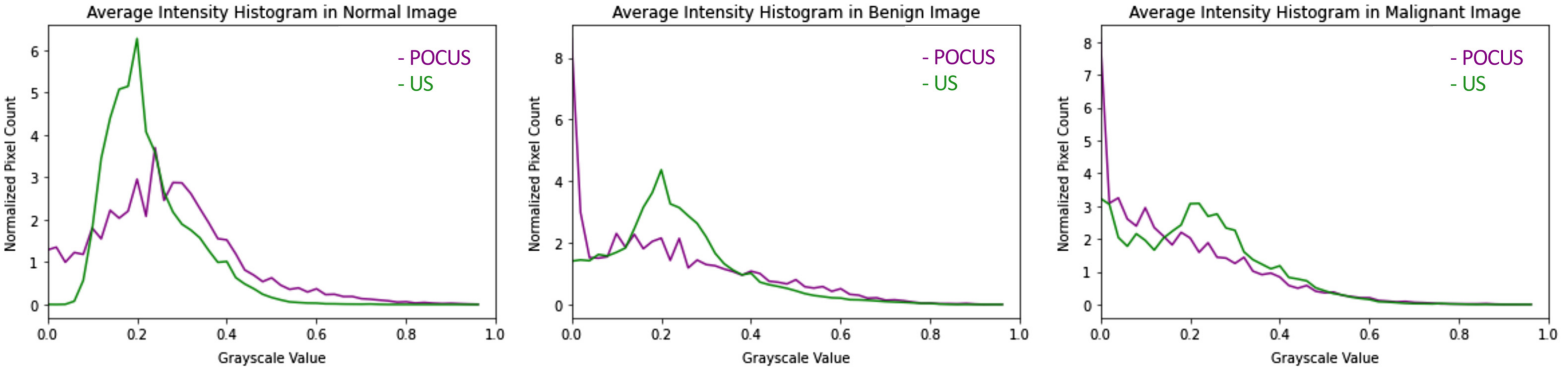
Histograms over the amount of pixels for each grayscale value for POCUS and standard US.^12^

Due to the usage of two different types of US machines, standard US, and POCUS, the appearances of the images in the two data sets differ. Figure 2 displays examples of images from each class collected with standard US and POCUS. There are a few differences between the images acquired with POCUS compared with standard US. First, the images collected with the POCUS device appeared to contain more noise compared with the standard US images. Second, the POCUS images contained more pixels in the darker range. This was shown by Sahlin,^12^ by comparing histograms of the pixel intensities between images from the two datasets, see Fig. 3.

Both the standard US and POCUS data sets were used for training, with the exception of 531 POCUS images allocated for testing. Table 1 displays the sizes of the training and test data sets. To avoid overfitting to a certain data set and biased results, it is important that all images from a patient are placed in the same set. Therefore, all images from each patient were either included in the test set or in the training set.

**Table 1:** Total number of images of each class for each of the two datasets.

	Standard US	POCUS	
	Train	Train	Test
Normal	386	463	284
Benign	254	173	131
Malignant	520	178	116
Total	1160	814	531

### 2.2 Preprocessing

Prior to using the data sets, some preprocessing of the images was performed. The images in both data sets were cropped to remove metadata near the edges. All images were zero-padded to be quadratic. Both the cropping and the zero padding were performed automatically using MATLAB. The resulting images were manually inspected. A few images in the POCUS test set became poorly cropped, for example, in some cases, the majority of the present lesion was removed. In such cases, the images were manually cropped. After cropping and zero padding, the POCUS images no longer contained any annotations from radiologists, whereas some of the standard US images still contained annotations. The latter will be discussed shortly in Sec. 3.

### 2.3 CycleGAN

A generative adversarial network (GAN) is a network consisting of two parts, a generator and a discriminator, which compete with each other during training.^13^ The generator strives to generate output that is as close as possible to real images from the domain, whereas the discriminator tries to distinguish the real training data from the synthetic data generated by the generator. The main usage of GAN is to generate unseen data with variation. However, in medical applications, generating unseen data can be hazardous because there is a risk that high-stake details are generated wrongly. Another way to generate unseen data without generating new images from scratch is to translate data from one image domain into another image domain. An example is translating standard US images into POCUS images, which preferably would conserve important structures. This can be achieved with a CycleGAN,^8^ an architecture that uses generators and discriminators similarly as in a GAN, but in this case, two generators and two discriminators are used. The CycleGAN generator, which transforms standard US images into POCUS images, was used to transform all the standard US images in Table 1 to appear as POCUS images. In this study, the original PyTorch implementation of CycleGAN^8^ has been used. Two different approaches were tried out. First, one CycleGAN model was trained for all image classes, and second, three separate CycleGAN models were trained, one for each image class. These will be referred to as ${\text{CycleGAN}}_{1}$ and ${\text{CycleGAN}}_{3}$, respectively. The standard US and POCUS train data sets presented in Table 1 were used to train the CycleGAN models. The models were trained for 200 epochs.

### 2.4 Classification Network

**Fig. 4:**
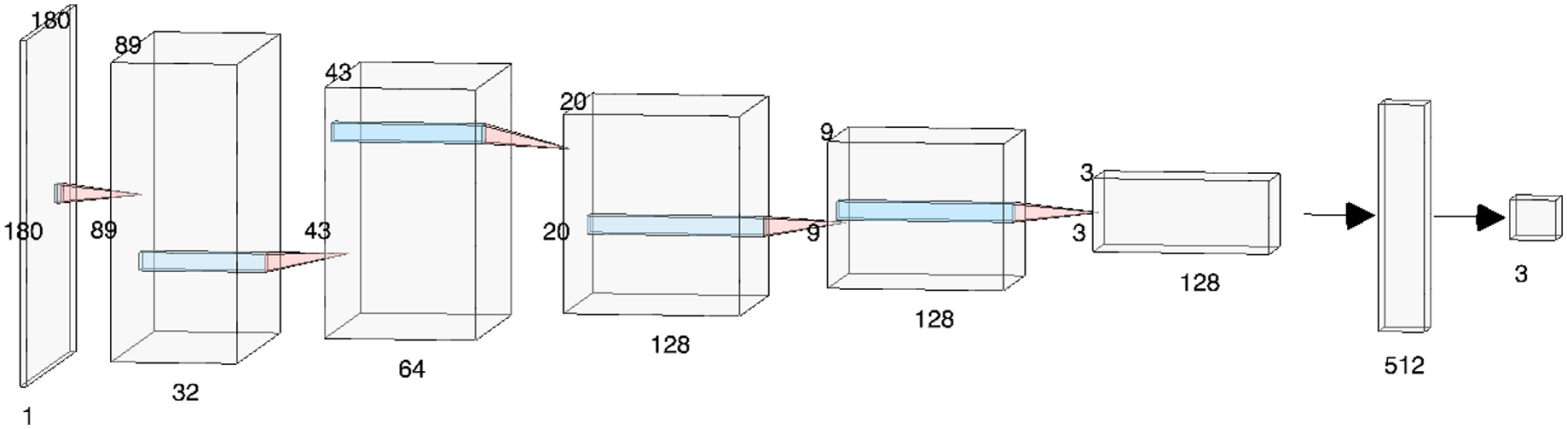
Architecture of the CNN used for classification. Image made in NN-SVG.^15^

A CNN was trained to classify breast POCUS images. The chosen architecture is based on a previously presented CNN,^10^ with the only difference of using one-channel images as input instead of three-channel images. The CNN was implemented in Python using the Keras library.^14^ The CNN consisted of five convolutional layers all with filter size of 3×3. The number of filters for each layer was 32, 64, 128, 128, and 128. Each convolutional layer was followed by a max pooling layer with pool size 2×2 and stride 2×2. The output from the final convolutional layer was flattened and used as input to the final two fully connected layers of sizes 512 and 3. The rectified linear unit activation function was used for all layers except for the final fully connected layer, where the softmax function was used instead. A dropout of 20% was added after the activation function for each convolutional layer. In addition, a dropout of 50% was added both after the last max pooling layer and after the first fully connected layer. The input size of the CNN was one-channel images of size 180×180. The architecture of the final CNN is displayed in Fig. 4.

### 2.5 Histogram Matching

Histogram matching can be used to transform an image such that the histogram of the pixel intensities matches a specified histogram. In this study, histogram matching was performed to make the images collected with the two different US modalities more similar. For each class, a histogram based on all POCUS training images belonging to the class was made. Each standard US image was transformed so that its histogram matched the POCUS histogram for the corresponding class. Histogram matching was implemented using the pre-built function^16^ from scikit-image. The original implementation performs histogram matching between two images. Some modifications were done to perform histogram matching between a histogram and an image.

### 2.6 Histogram Equalization

Histogram equalization is a method where the contrast of an image is improved by analyzing the histogram over all pixel intensities and spreading out the intensities equally. As the images acquired with the two different US modalities differed in their distributions of pixel intensities, histogram equalization could be of particular interest. The implementation from scikit-image was used.

### 2.7 Augmentation

Three different approaches with augmentation were investigated using the classification network described above. Both (1) spatial augmentation and (2) brightness/noise augmentation, and (3) the combination were evaluated. The POCUS training data combined with the standard US data were used during training, and the POCUS test set was used for evaluation. All augmentation was applied randomly for each image and each epoch using Keras. Each augmentation approach is described below.

#### 2.7.1 Spatial augmentation

The images were zoomed, shear transformed, and shifted horizontally and vertically, all within a range of 10%. They were also randomly flipped horizontally and randomly rotated within a range of 30 deg.

#### 2.7.2 Brightness/noise augmentation

The images’ brightness was randomly set between 70% and 130% of the original brightness. The brightness level under 100% resulted in a brighter image and over 100% resulted in a darker image. Gaussian noise was randomly applied to each pixel from a distribution with a mean of 0 and a variance of 0.01.

#### 2.7.3 Spatial and brightness/noise augmentation

The combined augmentation was implemented using both approaches described above.

### 2.8 Training Settings

The classification network described in Sec. 2.4 was trained using ADAM optimizer^17^ with the learning rate set to 0.0001 and otherwise default parameters. As the classification task contains multiple classes, the categorical cross-entropy loss was used during training. The loss was weighted so that all classes had an equal impact on the training. Each network was trained for 50 epochs and with a batch size of 32. The classification network architecture was trained on 16 different combinations of data according to the following list.

1. solely POCUS images (814 images)
2. POCUS and standard US images (1974 images)
3. POCUS and histogram matched standard US images (1974 images)
4. POCUS and standard US images with histogram equalization (1974 images)
5. POCUS images and synthetic POCUS images generated by ${\text{CycleGAN}}_{1}$ (1974 images)
6. POCUS images and synthetic POCUS images generated by ${\text{CycleGAN}}_{3}$ (1974 images)
7. POCUS images, standard US images, and synthetic POCUS images generated by ${\text{CycleGAN}}_{1}$ (3134 images)
8. POCUS images, standard US images, and synthetic POCUS images generated by ${\text{CycleGAN}}_{3}$ (3134 images)
9. POCUS images with random augmentation (814 images)
10. POCUS and standard US images, with random augmentation (1974 images)
11. POCUS and histogram matched standard US images with random augmentation (1974 images)
12. POCUS and standard US images with histogram equalization and random augmentation (1974 images)
13. POCUS images and synthetic POCUS images generated by ${\text{CycleGAN}}_{1}$, with random augmentation (1974 images)
14. POCUS images and synthetic POCUS images generated by ${\text{CycleGAN}}_{3}$, with random augmentation (1974 images)
15. POCUS images, standard US images, and synthetic POCUS images generated by ${\text{CycleGAN}}_{1}$, with random augmentation (3134 images)
16. POCUS images, standard US images, and synthetic POCUS images generated by ${\text{CycleGAN}}_{3}$, with random augmentation (3134 images).

The best-performing augmentation approach from Sec. 2.7 was applied to the classification networks mentioned above that include augmentation.

### 2.9 Evaluation of Performance

The quality of the images generated by the two different CycleGAN approaches was evaluated by calculating the Fréchet inception distance (FID) to the POCUS training data. For comparison, the FID was also calculated for the standard US data. The implementation of FID by Parmar et al.^18^ was used.

The evaluation of the classification networks presented in Sec. 2.8 was performed on the POCUS test set. The classification networks were evaluated by computing the receiver operating characteristic (ROC) curve and the area under the ROC curve (AUC). As the classification in this project is a multi-class problem with three classes (i.e., normal, benign, and malignant), the AUC was implemented by comparing one class against the other two classes. It was chosen to compare the malignant class (cancerous) with the normal and benign classes combined (non-cancerous). This choice was made to put the focus on not missing malignant tumors instead of always being correct in the non-cancerous classes. Furthermore, the sensitivity and specificity of the malignant class versus the others were evaluated. The threshold for finding the sensitivity and specificity was chosen automatically such that the Youden index^19^ was fulfilled

J=max(Sensitivity+Specificity−1). (1)

As detecting cancers is important, the Youden index was modified with an additional requirement of the sensitivity being at least 90%. Furthermore, a 95% confidence interval was estimated for both the sensitivity and the specificity by using bootstrap, i.e., randomly picking the POCUS test set with replacement 1000 times, and selecting the sensitivity and specificity at the automatically obtained threshold.

To evaluate the performance of all three classes, the balanced accuracy was calculated by taking the average over the sensitivities for each class. Furthermore, the 95% confidence interval of the AUC and balanced accuracy were estimated by using Bootstrap on the POCUS test set 1000 times. Statistical significance between the classification networks was calculated with a DeLong test on the AUCs followed by a correction for multiple comparisons with the Holm–Bonferroni method. The level of significance was set to 5%.

## 3 Results

### 3.1 CycleGAN

The FIDs between the POCUS images generated by the two different CycleGAN approaches and the POCUS training images are displayed in Table 2. The table also shows the FID between the standard US images and the POCUS training images. The distance for the standard US images is larger compared with the POCUS images generated with the CycleGAN approaches. This result is expected because the CycleGANs were trained to shift the domain of the standard US images into appearing as POCUS images.

**Table 2:** FIDs for the standard US images and CycleGAN-generated POCUS images versus POCUS images. A lower value means higher similarity.

Data	FID ↓
US	80.1
${\text{CycleGAN}}_{1}$	43.5
${\text{CycleGAN}}_{3}$	52.7

**Fig. 5:**
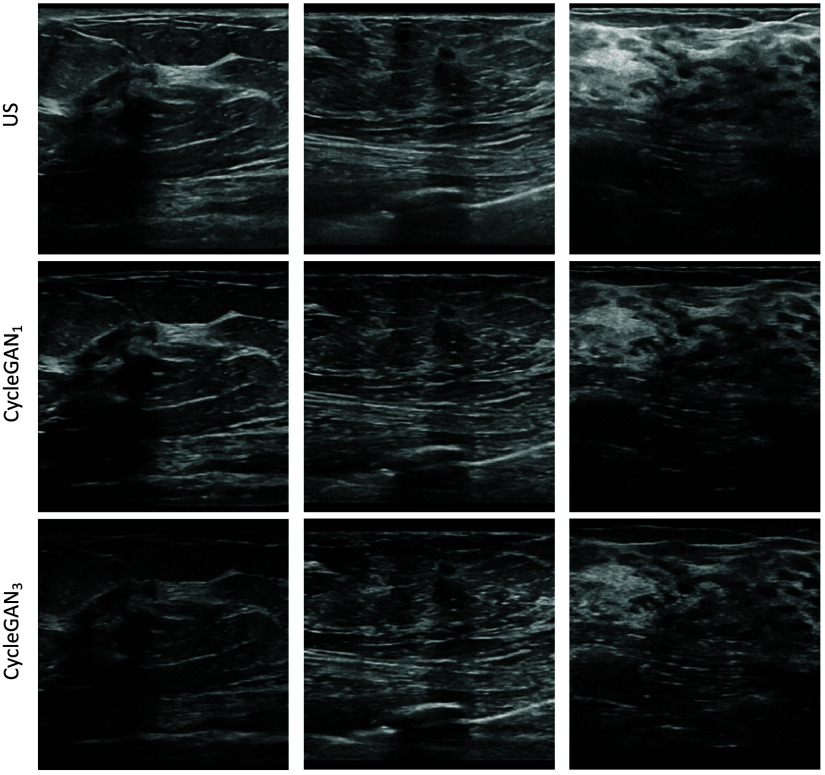
Examples of successfully generated POCUS images with both CycleGAN approaches.

Figure 5 displays three examples of standard US images translated by ${\text{CycleGAN}}_{1}$ and ${\text{CycleGAN}}_{3}$ into POCUS images. The CycleGAN-generated images appear a bit darker, which is expected because POCUS images in general contain more dark pixels than standard US.

**Fig. 6:**
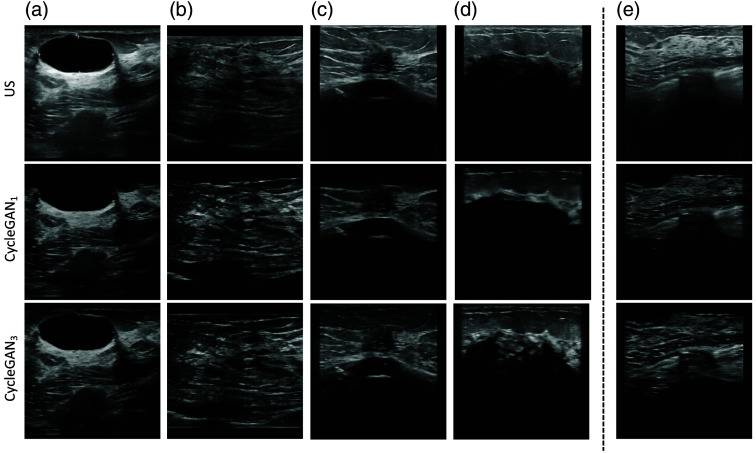
Panels (a)–(d) display cases where the ${\text{CycleGAN}}_{1}$-generated POCUS images contained darkened areas. Panel (e) shows an example where both the CycleGAN approaches contained darkened areas.

The POCUS images generated with ${\text{CycleGAN}}_{1}$ did at times include darkened areas, see in Figs. 6(a)–6(d). This is not desired because important information in the images is potentially lost. The same issue occurred for ${\text{CycleGAN}}_{3}$, see Fig. 6(e), but it was much more frequent among the ${\text{CycleGAN}}_{1}$-generated images.

**Fig. 7:**
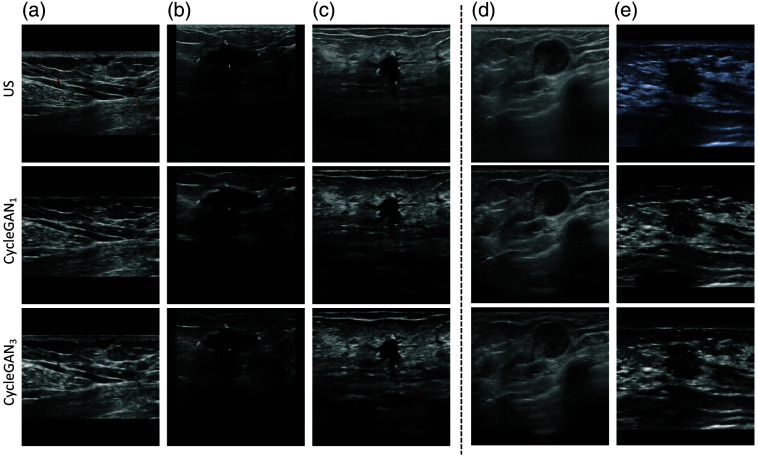
Example of vanishing annotation in CycleGAN-generated images is shown in panel (a), and examples of non-vanishing annotations are displayed in panels (b) and (c). Panels (d) and (e) show examples of artifacts. In panel (d), both the ${\text{CycleGAN}}_{1}$- and ${\text{CycleGAN}}_{3}$-generated POCUS images contain artifacts in the lower right part. In panel (e), the artifact is only appearing in the ${\text{CycleGAN}}_{1}$-generated POCUS image as a dark structure at the bottom.

An interesting property of both the CycleGAN approaches is the vanishing of annotations such as text or symbols. This property can be seen in Fig. 7(a), where the original standard US image contains markings in the center of the image, which are no longer present in the corresponding CycleGAN-generated POCUS images. However, for some of the translated images, the annotations were still present; examples of such images are shown in Figs. 7(b) and 7(c).

A problem with the CycleGAN-generated images is that sometimes artifacts appear. The CycleGAN-generated POCUS images are labeled with the same label as the standard US images they have been translated from. In such cases, where artifacts appear, the label might no longer be correct. Figure 7(d) shows an example of artifacts, where the standard US image contains shadowing in the lower right part, which could be perceived as a round smooth anechoic structure with a posterior hyperechoic rim in both the ${\text{CycleGAN}}_{1}$- and ${\text{CycleGAN}}_{3}$-generated images. The contrast of all the images in this column was decreased for visualization purposes, making the artifact appear more clearly. Another example of an artifact is displayed in Fig. 7(e), where ${\text{CycleGAN}}_{1}$ contains a dark oval-shaped artifact in the bottom part of the image.

### 3.2 Classification

Table 3 displays the results of applying different types of augmentation. The augmentation including brightness and noise was significantly worse than using spatial augmentation or a combination of spatial, brightness, and noise augmentation with corresponding p-values of $6.5\cdot 10^{-7}$ and $4.9\cdot 10^{-8}$. No significant difference between spatial augmentation and the combination of spatial, brightness, and noise could be found. For simplicity, it was chosen to use spatial augmentation during the training for the combinations of data where augmentation was applied.

**Table 3:** AUC performance of the classification network when trained on POCUS and standard US data with applied spatial augmentation (spatial aug) and/or brightness and noise augmentation (brightness/noise aug).

Spatial aug	Brightness/noise aug	AUC (%)	AUC 95% CI (%)
x	—	94.9	92.7 to 96.6
—	x	84.6	79.9 to 88.6
x	x	94.3	92.4 to 96.2

**Fig. 8:**
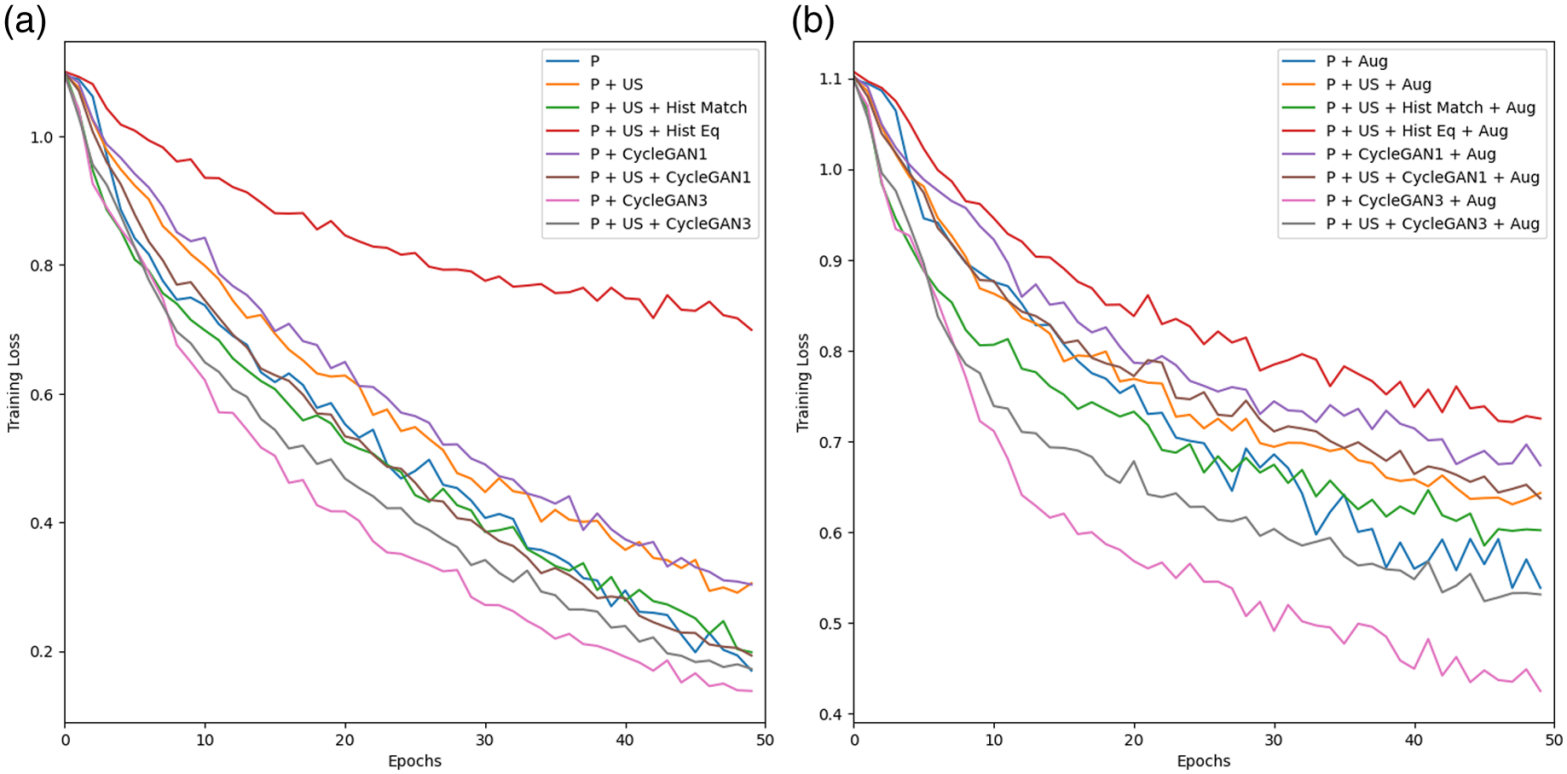
Training loss curves for the classification networks trained without augmentation (a) and with augmentation (b).

Figure 8 shows the training loss for all the classification networks. The network trained on the combination of POCUS and standard US images with applied histogram equalization with/without augmentation has a less steep decrease compared with the other networks.

The performance of the classification network trained on each combination of data is shown in Table 4. Adding spatial augmentation improves the AUC performance for most of the classification networks. The accuracy values are in a lower range compared with the values for the AUC. The accuracy is based on three classes, whereas the AUC is based on only two; thus, the accuracy metric is used for the more complex problem.

**Table 4:** Results for the balanced accuracy (ACC) and AUC, obtained by training the classification network on each combination of data. Acronyms: POCUS (P), standard ultrasound (US), matching the histogram of standard ultrasound images to POCUS images (Hist. Match), histogram equalization applied to training and test data (Hist. Eq.), standard ultrasound images converted into POCUS by one of the CycleGANs (${\text{CycleGAN}}_{1}$, ${\text{CycleGAN}}_{3}$), and spatial augmentation applied to training data (Aug).

Combination of data	ACC (%)	ACC 95% CI (%)	AUC (%)	AUC 95% CI (%)
P	60.8	56.6 to 65.2	75.0	69.4 to 80.0
P + US	68.3	64.0 to 72.6	88.2	84.7 to 91.6
P + US + Hist. Match	64.6	60.0 to 69.1	77.4	72.1 to 82.7
P + US + Hist. Eq.	61.7	57.9 to 65.7	93.9	91.8 to 95.8
P + ${\text{CycleGAN}}_{1}$	66.0	61.7 to 69.9	90.5	87.3 to 93.5
P + ${\text{CycleGAN}}_{3}$	74.7	71.3 to 78.2	95.3	93.4 to 97.0
P + US + ${\text{CycleGAN}}_{1}$	69.8	66.1 to 73.3	92.6	89.9 to 94.8
P + US + ${\text{CycleGAN}}_{3}$	71.2	67.1 to 75.1	90.7	87.8 to 93.5
P + Aug	62.9	59.0 to 66.8	92.3	89.7 to 94.5
P + US + Aug	67.4	63.2 to 71.5	94.9	92.7 to 96.6
P + US + Hist. Match + Aug	64.1	59.9 to 68.1	88.1	83.5 to 92.2
P + US + Hist. Eq. + Aug	63.3	59.3 to 67.6	93.4	90.9 to 95.5
P + ${\text{CycleGAN}}_{1}$ + Aug	67.7	63.7 to 71.6	94.7	92.4 to 96.6
P + ${\text{CycleGAN}}_{3}$ + Aug	69.8	65.9 to 73.9	94.1	91.9 to 96.2
P + US + ${\text{CycleGAN}}_{1}$ + Aug	67.3	63.1 to 71.2	95.0	93.0 to 96.8
P + US + ${\text{CycleGAN}}_{3}$ + Aug	68.8	65.0 to 72.6	94.6	92.5 to 96.5

Table 5 displays the sensitivity and specificity of each classification network. For almost all the networks, the specificity increases when using spatial augmentation.

**Table 5:** Results for sensitivity (Sens) and specificity (Spec), obtained by training the classification network on each combination of data. Acronyms: POCUS (P), standard ultrasound (US), matching the histogram of standard ultrasound images to POCUS images (Hist. Match), histogram equalization applied to training and test data (Hist. Eq.), standard ultrasound images converted into POCUS by one of the CycleGANs (${\text{CycleGAN}}_{1}$, ${\text{CycleGAN}}_{3}$), and spatial augmentation applied to training data (Aug).

Combination of data	Sens (%)	Sens 95% CI (%)	Spec (%)	Spec 95% CI (%)
P	95.7	91.7 to 99.1	20.7	16.5 to 24.6
P + US	90.5	85.2 to 95.5	61.7	57.1 to 66.7
P + US + Hist. Match	90.5	84.8 to 95.5	37.1	32.3 to 41.7
P + US + Hist. Eq.	94.0	89.3 to 98.2	78.1	74.2 to 82.4
P + ${\text{CycleGAN}}_{1}$	91.4	86.3 to 96.5	71.1	66.4 to 75.4
P + ${\mathrm{CycleGAN}}_{3}$	91.4	85.6 to 96.4	88.4	85.1 to 91.1
P + US + ${\text{CycleGAN}}_{1}$	90.5	84.9 to 95.7	79.3	75.2 to 83.0
P + US + ${\text{CycleGAN}}_{3}$	91.4	86.0 to 96.2	70.4	65.8 to 74.6
P + Aug	91.4	86.1 to 96.1	77.8	73.6 to 81.6
P + US + Aug	93.1	88.1 to 97.5	83.9	80.0 to 87.2
P + US + Hist. Match + Aug	90.5	84.3 to 95.2	71.1	66.8 to 75.3
P + US + Hist. Eq. + Aug	90.5	84.6 to 95.3	82.9	79.0 to 86.5
P + ${\text{CycleGAN}}_{1}$ + Aug	94.0	89.3 to 98.2	84.1	80.4 to 87.5
P + ${\text{CycleGAN}}_{3}$ + Aug	93.1	87.6 to 97.3	77.3	73.5 to 81.5
P + US + ${\text{CycleGAN}}_{1}$ + Aug	93.1	88.0 to 97.4	81.0	76.9 to 84.6
P + US + ${\text{CycleGAN}}_{3}$ + Aug	93.1	88.0 to 97.4	84.1	80.3 to 87.4

**Fig. 9:**
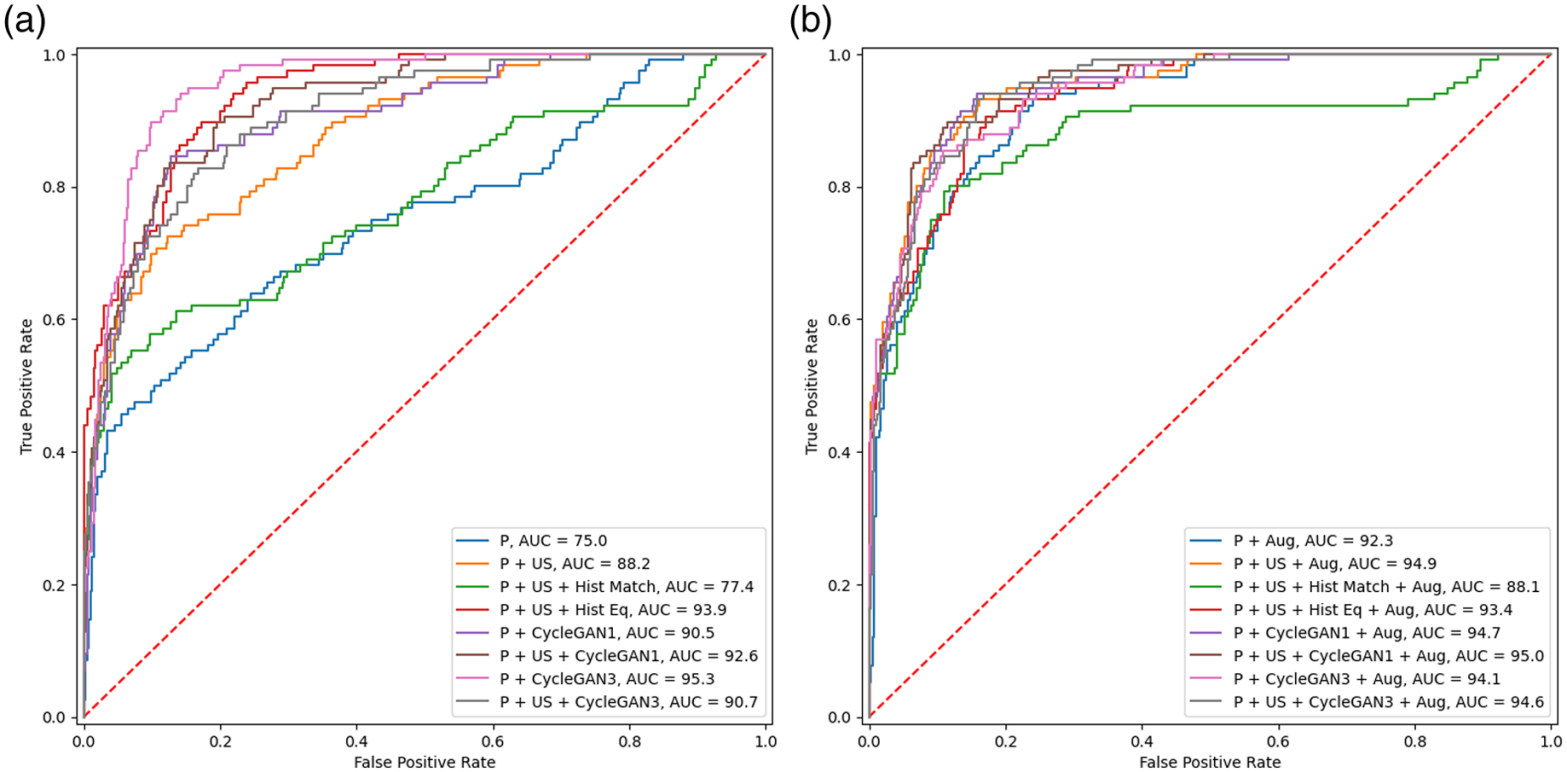
ROC curves for the classification networks trained without augmentation (a) and with augmentation (b).

Figure 9 shows the ROC curves for all the classification networks. The curve of the classification network trained on solely POCUS images and the network trained on POCUS and standard US images with histogram matching are inferior to training on the other combinations of data. The curves for the networks trained with applied augmentation are more equal compared with the ones where no augmentation is applied.

The results from the significance test between the classification network AUCs are shown in Table 6. All classification networks, except the network trained on POCUS and standard US images with histogram matching, are significantly better (p<0.01) than solely using POCUS images during training. There are several classification networks where no significant difference could be found. Furthermore, none of the classification networks is significantly better than all others. Applying augmentation either leads to significantly better results or no significant difference could be found compared with training without augmentation.

**Table 6:** Results for the DeLong test with correction for multiple comparisons using the Holm–Bonferroni method. The significant difference between the two networks is indicated by * (p<0.05) or ** (p<0.01). Acronyms: POCUS (P), standard ultrasound (US), matching the histogram of standard to POCUS (Hist. Match), histogram equalization applied to training and test data (Hist. Eq.), standard ultrasound images converted into POCUS by one of the CycleGANs (${\text{CycleGAN}}_{1}$, ${\text{CycleGAN}}_{3}$), and spatial augmentation applied to training data (Aug).

	1	2	3	4	5	6	7	8	9	10	11	12	13	14	15	16
1. (P)	—	**	—	**	**	**	**	**	**	**	**	**	**	**	**	**
2. (P + US)	**	—	**	—	—	**	*	—	—	**	—	—	**	**	**	**
3. (P + US + Hist. Match)	—	**	—	**	**	**	**	**	**	**	**	*	**	**	**	**
4. (P + US + Hist. Eq.)	**	—	**	—	—	**	—	—	—	**	—	—	**	*	**	**
5. (P + ${\mathrm{CycleGAN}}_{1}$)	**	—	**	—	—	*	—	—	—	—	—	—	—	—	*	—
6. (P + ${\text{CycleGAN}}_{3}$)	**	**	**	**	*	—	—	**	—	—	—	**	—	—	—	—
7. (P + US + ${\text{CycleGAN}}_{1}$)	**	*	**	—	—	—	—	—	—	—	—	—	—	—	—	—
8. (P + US + ${\text{CycleGAN}}_{3}$)	**	—	**	—	—	**	—	—	—	—	—	—	—	—	*	—
9. (P + Aug)	**	—	**	—	—	—	—	—	—	—	—	—	—	—	—	—
10. (P + US + Aug)	**	**	**	**	—	—	—	—	—	—	—	**	—	—	—	—
11. (P + US + Hist. Match + Aug)	**	—	**	—	—	—	—	—	—	—	—	—	—	—	—	—
12. (P + US + Hist. Eq. + Aug)	**	—	*	—	—	**	—	—	—	**	—	—	**	**	**	**
13. (P + ${\text{CycleGAN}}_{1}$ + Aug)	**	**	**	**	—	—	—	—	—	—	—	**	—	—	—	—
14. (P + ${\text{CycleGAN}}_{3}$ + Aug)	**	**	**	*	—	—	—	—	—	—	—	**	—	—	—	—
15. (P + US + ${\text{CycleGAN}}_{1}$ + Aug)	**	**	**	**	*	—	—	*	—	—	—	**	—	—	—	—
16. (P + US + ${\text{CycleGAN}}_{3}$ + Aug)	**	**	**	**	—	—	—	—	—	—	—	**	—	—	—	—

## 4 Discussion

When ${\text{CycleGAN}}_{1}$ is trained using examples from all classes simultaneously, common structures from one class may be inserted in images from another class. This could lead to the appearance of artifacts because the network is not trained to distinguish between the classes. Artifacts could be problematic when those images later are used to train a classification network because the label of some images might have changed due to such artifacts. To avoid this issue, three separate CycleGANs were trained, one for each class (${\text{CycleGAN}}_{3}$). Hence, the generator should learn the properties of a specific class and hopefully not generate images containing properties from different classes. However, our results show that artifacts still appear, see Fig. 7(d), although less frequently.

Training three separate CycleGANs, one for each label, did only partially solve the artifact issue. Another alternative could be to investigate the possibility of adding a constraint to the CycleGAN. The constraint should make sure that the translated image does not differ too much from the original image. For example, Yang et al.^20^ proposed a structure-constrained CycleGAN used to translate brain MR images to CT and received promising results.

Figure 7 shows examples of how standard US images containing annotations are affected when translated into POCUS images with CycleGAN. In panel (a), the annotation vanishes in both the ${\text{CycleGAN}}_{1}$- and ${\text{CycleGAN}}_{3}$-generated images; however, in panels (b) and (c), the annotations still exist but are blurred out. US images are in grayscale, but their annotations can sometimes be in color. The annotation in the first column is colored red, a color not present in the POCUS domain; hence, it should not be appearing in the CycleGAN-generated POCUS image. However, the annotations in panels (b) and (c) are of the color white, a color existing in the grayscale domain, which makes it possible to be a feature in the POCUS domain and not as simple to rule out as not a POCUS feature.

The end goal of this work is to detect cancer, i.e., being able to distinguish malignant from normal and benign. Hence, the AUC, which is a more robust measurement than accuracy because it is not dependent on a specific classification threshold, will be used for comparison of the results in Table 4. It is shown that all classification networks are performing better compared with only training the network on POCUS images. This is supported by Table 6, where the classification network trained on solely POCUS images is significantly worse than almost all other networks. This indicates that adding more data will increase the classification performance.

Both CycleGAN approaches achieved a better FID to the POCUS training data compared with standard US, see Table 2. This indicates that the CycleGAN-generated POCUS images are more similar to the POCUS images compared with standard US images. The eight classification networks trained with CycleGAN-generated POCUS images performed better compared with training on POCUS images combined with standard US images. Six out of these networks, networks 6, 7, 13, 14, 15, and 16 in Table 6 performed significantly better with corresponding corrected p-values of $6.5\cdot 10^{-5}$, $2.6\cdot 10^{-2}$, $1.2\cdot 10^{-3}$, $3.6\cdot 10^{-3}$, $6.7\cdot 10^{-4}$, and $5.6\cdot 10^{-4}$. Furthermore, Table 5 shows that all networks, which include CycleGAN-generated POCUS images, had higher specificity compared with the network trained on the combination of POCUS and standard US images. However, applying augmentation to the network trained on POCUS and standard US images increases the specificity to the same level as for the networks trained with CycleGAN-generated images. This is corroborated by Table 6, which shows that when augmentation is applied to the network trained on the combination of POCUS and standard US images, the statistical testing failed to find a significant difference to the networks trained with CycleGAN-generated POCUS images. Furthermore, this can be seen in Fig. 9 where the ROC curve for the network trained on the combination of POCUS and standard US images is getting closer to the curves of the networks including CycleGAN when augmentation is applied compared with when it is not applied.

Using histogram matching without applied augmentation was significantly worse than almost all networks. The histogram matching was performed for each class by finding the histogram of all POCUS images and then using these to shift the distribution of pixel intensities of the US images. An issue with this is that within each class, the histograms for each image could vary. For example, images containing benign lesions will differ in distributions of pixel intensities depending on the size of the lesion. Hence, using one histogram for the whole class is not appropriate.

Training the network on POCUS and ${\text{CycleGAN}}_{3}$-generated POCUS images was significantly better (AUC 95% CI: 93.4 to 97.0) than the networks 1, 2, 3, 4, 5, and 8 in Table 6, with corresponding p-values $4.9\cdot 10^{-12}$, $6.5\cdot 10^{-5}$, $1.9\cdot 10^{-9}$, $5.3\cdot 10^{-4}$, $2.2\cdot 10^{-2}$, and $5.6\cdot 10^{-3}$. The first CycleGAN approach, ${\text{CycleGAN}}_{1}$, did contain dark areas, which contributed to information lost in the image and artifacts, see Figs. 6 and 7(e). This could be the reason behind the performance results of the network using POCUS images combined with ${\text{CycleGAN}}_{1}$-generated POCUS images being significantly (p<0.05) different than the network using ${\text{CycleGAN}}_{3}$-generated POCUS images. However, when adding standard US images and/or applying augmentation, it failed to find any significant difference between the two CycleGAN approaches.

For all classification networks in Table 6, applying augmentation was significantly better, or no significant difference was found compared with not applying augmentation. Hence, it is an effective way of increasing the amount of training data, potentially improving performance. In Table 5, it is shown that for almost all classification networks, it was possible to achieve an increased specificity when augmentation was applied.

The labels of the POCUS images were determined based on standard-of-care practices and histological analysis. However, a potential limitation is the lack of a 1-year follow-up for these cases. Nonetheless, the clinical routine in Sweden mitigates this concern by routinely performing biopsies on all indeterminate or suspicious findings, as well as some benign lesions. For instance, BI-RADS^21^ three lesions are typically biopsied rather than managed with short-term follow-ups. As a result, the likelihood of incorrect false-negative labels being a significant source of error is minimal.

## 5 Conclusion

In this work, a unique data set containing breast US images collected with POCUS was used. A comparison and extension of some state-of-the-art methods utilizing different combinations of data during training was performed for improved performance for POCUS image classification to gain knowledge on how to handle the scarcity of data. The results indicate that more images and increased variability within the data set improve the performance of the classification network. Performing random spatial augmentation to the images improves the results but only with a significant improvement in some of the cases. Furthermore, two different approaches using CycleGAN were implemented, first by training one CycleGAN for all labels and second by training three CycleGANs, i.e., one for each label. The images generated with CycleGAN positively impact the performance of the classification network compared with only training with POCUS images. However, it could not be statistically shown that the CycleGAN approach would be better than adding standard US images and applying spatial augmentation. Almost all the methods used for expanding the training data significantly improve the classification network compared with only using POCUS images during training. Overall, the results are promising for an accessible breast diagnostic tool in low-resource settings.

## Data Availability

Sharing the data is not allowed due to the current ethical approval. The code for the CycleGAN is available from the original paper;^8^ the remaining code and trained classification networks can be shared upon reasonable request.
